# A butterfly with olive green eyes discovered in the United States and the Neotropics (Lepidoptera, Lycaenidae, Eumaeini)

**DOI:** 10.3897/zookeys.305.5081

**Published:** 2013-05-28

**Authors:** Robert K. Robbins, Jeffrey Glassberg

**Affiliations:** 1 Department of Entomology, Smithsonian Institution, PO Box 37012, NHB Stop 105 (E-514), Washington, DC 20013-7012 USA; 2 North American Butterfly Association, 4 Delaware Rd., Morristown, NJ 07960, USA

**Keywords:** Butterfly Eye Color, Curaçao, Isla Margarita, *Ministrymon azia*, *Ministrymon janevicroy*, Vicroy’s Ministreak

## Abstract

We describe *Ministrymon janevicroy* Glassberg, **sp. n.**, from the United States (Texas). Its wing pattern closely resembles that of the widespread and well-known lycaenid, *Ministrymon azia* (Hewitson). The new species is distinguished by the structure of its male and female genitalia, by the patterning of the ground color on the basal half of the ventral hindwing surface, and by the color of its eyes. Adults of *Ministrymon janevicroy* in nature have olive green eyes in contrast to the dark brown/black eyes of *Ministrymon azia*. *Ministrymon janevicroy* occurs in dry deciduous forest and scrub from the United States (Texas) to Costa Rica (Guanacaste) with disjunct populations on Curaçao and Isla Margarita (Venezuela). In contrast, *Ministrymon azia* occurs from the United States to southern Brazil and Chile in both dry and wet lowland habitats. Nomenclaturally, we remove the name *Electrostrymon grumus* K. Johnson & Kroenlein, 1993, from the synonymy of *Ministrymon azia* (where it had been listed as a synonym of *Ministrymon hernandezi* Schwartz & K. Johnson, 1992). We accord priority to *Angulopis hernandezi* K. Johnson & Kroenlein, 1993 over *Electrostrymon grumus* K. Johnson & Kroenlein, 1993, **syn. n.**, which currently is placed in *Ziegleria* K. Johnson, 1993. The English name Vicroy’s Ministreak is proposed for *Ministrymon janevicroy*. We update biological records of dispersal and caterpillar food plants, previously attributed to *Ministrymon azia*, in light of the new taxonomy.

## Introduction

**Figure 1.**
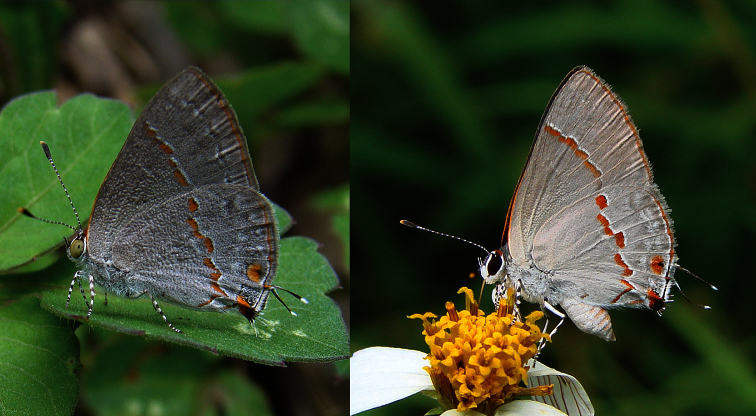
Olive green eyes of *Ministrymon janevicroy* (left, Orizaba, Veracruz, Mexico) and the dark brown/black eyes of *Ministrymon azia* (Chavarrillo, Veracruz, Mexico).

*Ministrymon azia* (Hewitson) (Fig. 1) is widely cited in faunal lists and occurs from the southern United States to southern Brazil, Paraguay, and Argentina in virtually all lowland habitats, ranging from desert in coastal Peru and Chile to rainforest in the Amazon Basin (Godman and Salvin 1887-1901, Draudt 1919-1920, Kaye 1921, Talbot 1928, Holland 1931, Stallings and Turner 1946, Hayward 1958, Brown and Mielke 1967, Ebert 1970, Lamas 1977, Robbins et al. 1996, 2012a, Gareca et al. 2009, Duarte et al. 2010). Adults of *Ministrymon azia* appear to be highly dispersive, having been recorded migrating through Portachuelo Pass in northern Venezuela and being dispersed by dry season trade winds in Panama (Beebe 1951, Robbins and Small 1981). Caterpillars of *Ministrymon azia* eat the flowers of a wide variety of Fabaceae and are discussed in the biological control and agriculture literature (e.g., Cock 1985, Harley et al. 1995, Fernández and Rodríguez 1997, Vargas and Parra 2009). The ventral wing pattern of *Ministrymon azia* west of the Andes is slightly different from that in other parts of its range, but genitalic variation is negligible (Johnson and Miller 1991). The nomenclature and taxonomy of *Ministrymon azia* are stable (Robbins and Lamas 2002, ICZN 2006). This species lacks a common name in the agricultural literature (Bosik 1997) but has been called Gray Ministreak in recent works dealing with North American butterflies (Cassie et al. 1995, Wauer 2004, Allen et al. 2005, Cech and Tudor 2005).

The generic placement of *Ministrymon azia* is a bit of an historical puzzle. Clench (1961: 196) described *Ministrymon* based on the presence of “two small erect ventral teeth near the tip” of the penis, but placed *azia* in *Tmolus* Hübner, a genus that lacks these teeth (as noted by Clench 1961). *Ministrymon azia* has four small erect teeth (Figs 5–6, first illustrated by Johnson and Miller 1991). It is puzzling that Clench did not observe the teeth because they are reasonably conspicuous. Robbins (2004a) listed 22 species in *Ministrymon* primarily based upon the presence of teeth on the ventral side of the penis near the tip. These teeth are otherwise unreported in the Eumaeini.

We recently discovered that the traditional species concept of *Ministrymon azia* includes a cryptic species that occurs sympatrically and synchronically with *Ministrymon azia* from the United States (Texas) south into the Neotropics. The cryptic species was discovered in Texas and Mexico (Glassberg 2005, 2012) because its adults have olive green eyes instead of the dark brown/black eyes of *Ministrymon azia* (Fig. 1). So far as we are aware, this is the first time that an undescribed butterfly species has been recognized on the basis of eye color, a point that is amplified in the discussion. We subsequently found that the genitalia and ventral wing patterns of these species differ substantially and consistently. It is the purpose of this paper to give the undescribed species a scientific name, to present data that support the hypothesis that it is biologically distinct, and to update information on the biology of these species using the new taxonomy.

## Materials and methods

Standard methods were used to dissect genitalia and to prepare them for examination with an SEM (Robbins 1991). Genitalic terminology follows that in Clench (1961) and Klots (1970), as modified for the Eumaeini (Robbins 1991). Forewing length was measured with a digital vernier caliper. Wing vein names follow Comstock (1918), and terminology for male secondary sexual characters follows Robbins (1991) and Robbins et al. (2012b). Museum specimens of *Ministrymon janevicroy* studied are deposited (unless otherwise noted) in the USNM (see below for repository acronyms). Thirty images of *Ministrymon janevicroy* in nature from Texas, Mexico, and Venezuela were assembled (most taken without a flash). For this study, specimens and images of *Ministrymon janevicroy* were compared with a study series of 550+ specimens of *Ministrymon azia* from 20 countries deposited in the USNM and with 44 images of individuals of *Ministrymon azia* in nature.

We list genitalic dissections in Supplementary file 1 *Ministrymon* Genitalia Examined, images in nature in Supplementary file 2 Images of Live Butterflies, and data on forewing length and frequency of eye-color in Supplementary file 3 *Ministrymon janevicroy* Datasets.

Museum specimens cited in this study are deposited in the following collections – museum acronyms from Evenhuis (1993).

**AMNH** American Museum of Natural History, New York, USA

**BMNH** The Natural History Museum [formerly British Museum (Natural History) ], London, United Kingdom

**DZUP** Museu de Entomología Pe. Jesus Santiago Moure, Universidade Federal do Paraná, Curitiba, Paraná, Brazil

**FSMC** Florida Museum of Natural History (Allyn Museum/McGuire Center), University of Florida, Gainesville, Florida, USA

**MC** Personal collection of Alfred Moser, Sao Leopoldo, RS, Brazil

**TAMU** Texas A & M University, College Station, USA

**UCRC** Entomology Research Museum, University of California, Riverside, California, USA

**USNM** National Museum of Natural History, Smithsonian Institution, Washington, DC, USA

## Taxonomy

### Ministrymon janevicroy

Glassberg sp. n.

urn:lsid:zoobank.org:act:10ED3009-21F8-4B7C-B9B4-A93A5D867972

http://species-id.net/wiki/Ministrymon_janevicroy

Figs 1 –4 , 6 –9

#### Type material.

**Holotype:** ♂ (Fig. 3). [hand written in black India Ink on white paper] July 12, 1969/Santa Ana Ref.[uge]/Hidalgo Co[unty]/Texas/J.B. Sullivan. [printed red label] Holotype/*Ministrymon janevicroy*/Glassberg. [printed green label] Genitalia No./2013: 10♂/R. K. Robbins. Deposited USNM. **Paratypes** (9♂, 4♀). Uvalde County. **1**♂, Concan[,] Tex[as]/7[July]-6-[19]36/W.D. Field. Hidalgo County. **8**♂, same data as holotype. **1**♀, June 12, 1976/Sullivan City/Hidalgo Co./Texas/J.B. Sullivan. **1**♀ (Fig. 3), Pharr, Texas/20 April 1948/H.A. Freeman (via Nicolay collection). Kerr County. **2**♀, Kerrville/Jun[e] 1917/Texas (via Barnes Collection). Paratypes have a blue printed paratype label and are deposited USNM. Five paratypes have been dissected and labeled as such (cf. supplementary file).

#### Other specimens examined

(excluded from the type series). Mexico: 33♂♂, 3♀♀. El Salvador: 1♀. Nicaragua: 4♂♂, 6♀♀. Costa Rica: 4♂♂, 1♀. Curaçao 2♂♂, 5♀♀ (FSMC). Venezuela: 2♀.

#### Images in nature examined

(excluded from the type series, specifics listed in a supplementary file).United States (Texas): 19, Mexico 10, Venezuela 1.

#### Etymology.

This species is named for my wife, Jane Vicroy Scott, whose love and patient forbearance have sustained me, and made me a more effective advocate for butterflies. Her tireless work in support of the North American Butterfly Association, especially with the National Butterfly Center in the Rio Grande Valley (less than 40km from the type locality of *Ministrymon janevicroy*), has helped make the world a little bit more friendly for butterflies and thus for people. The name is a non-latinized noun in apposition. I have proposed the English name Vicroy’s Ministreak for this species (Glassberg 2012).

#### Diagnosis and description.

*Ministrymon janevicroy* is placed in *Ministrymon* because there are small erect teeth on the ventral surface of the penis near the distal end (Fig. 6). Clench (1961) originally noted this generic distinguishing trait, albeit limited to two teeth. In museum collections, specimens of *Ministrymon janevicroy* are routinely curated with *Ministrymon azia* because of the similarity in ventral wing patterns (Fig. 1). For this reason, we differentiate *Ministrymon janevicroy* from *Ministrymon azia*. However, *Ministrymon* has not been revised, so it would be premature to suggest that these species are phylogenetic sisters, even if it is likely.

Adults of *Ministrymon janevicroy* are differentiated from those of *Ministrymon azia* by (1) the male and female genitalia, (2) the ventral wing pattern, and (3) the color of the eyes.

The male genitalia of *Ministrymon janevicroy* (7 dissections, listed in supplementary information) differ consistently from those of *Ministrymon azia* (11 dissections), primarily by structures of the posterior penis (Fig. 6). The four—as illustrated—or five small erect teeth on the ventral surface of the penis tip of *Ministrymon janevicroy* are clustered anterior of the posterior penis tip while in *Ministrymon azia* two teeth are located near the posterior penis edge, well posterior of two other teeth. Inside the penis shaft, there is a single slender cornutus in *Ministrymon janevicroy* while the vesica on either side of the cornutus in *Ministrymon azia* is sclerotized. Depending upon the amount of sclerotization and the extent to which the vesica is everted, these sclerotizations may appear as a double prong (as in Fig. 6) or as a pair of lateral sclerotized triangular teeth. The shorter and squatter valvae in ventral aspect and the shallower and wider notch between the labides in dorsal aspect of *Ministrymon janevicroy* (illustrated in Fig. 6) represent individual variation and do not distinguish the species. The illustrated longer saccus of *Ministrymon janevicroy* (Fig. 6) may differentiate the species statistically, but this length in the study series was overlapping.

The female genitalia of *Ministrymon janevicroy* (6 dissections) differ substantially and consistently from those of *Ministrymon azia* (5 dissections). The female genitalia of *Ministrymon janevicroy* are distinguished from those of *Ministrymon azia* by a membranous “neck” just posterior of the cervix (arrow on the left of Fig. 7) and the lack of a well-formed posterior pouch from which the ductus seminalis arises (arrow on the right of Fig. 7). These differences are conspicuous and immediately distinguish the species. The illustrated ductus bursae of *Ministrymon janevicroy* is longer than that of *Ministrymon azia* (Fig. 7), but this difference represents individual variation.

**Figure 2.**
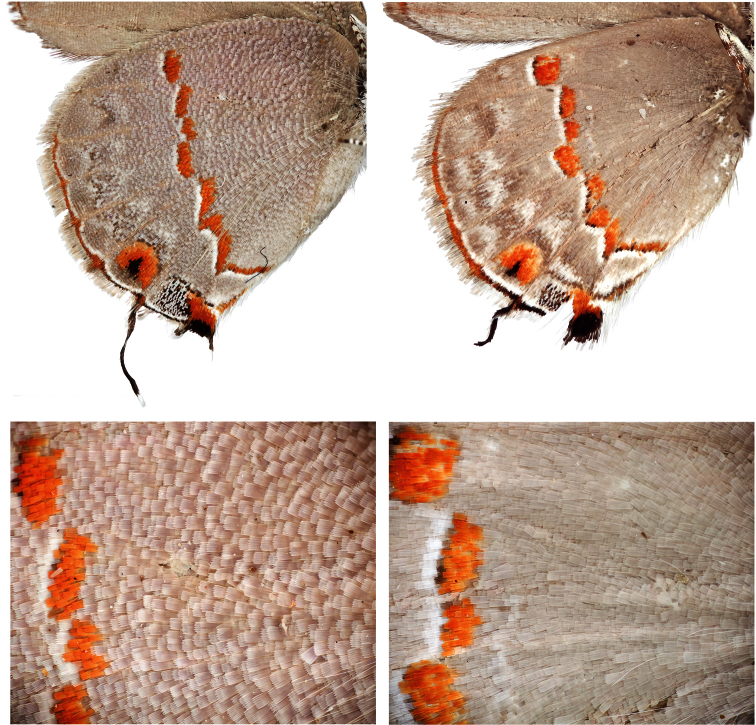
*Ministrymon janevicroy* (left, close-up on bottom) with variegated “pebbly-textured” ground color and *Ministrymon azia* (right) with “smooth-textured” gray appearance. Both specimens from Managua, Nicaragua.

Glassberg (2012) distinguished the variegated “pebbly-textured” appearance on the basal half of the ventral hindwing surfaces of *Ministrymon janevicroy* from the more “smooth-textured” appearance in *Ministrymon azia* (Fig. 2). In the study series, the variegated “pebbly-textured” appearance on the hindwing (but not always the forewing) correlates without exception with genitalic structures for the 29 dissected specimens of *Ministrymon janevicroy* and *Ministrymon azia*. The wing scales that are responsible for the variegated “pebbly-textured” appearance in *Ministrymon janevicroy* are gray basally and whitish at their tips and do not lie flat against the wing. In contrast, the wing scales that are responsible for the gray “smooth-textured” appearance in *Ministrymon azia* are almost uniformly gray and lie flat against the wings. The scales in *Ministrymon janevicroy* are also wider than those of *Ministrymon azia*, and have a jagged terminal edge, but it is unclear how these shape differences affect wing appearance.

**Figure 3.**
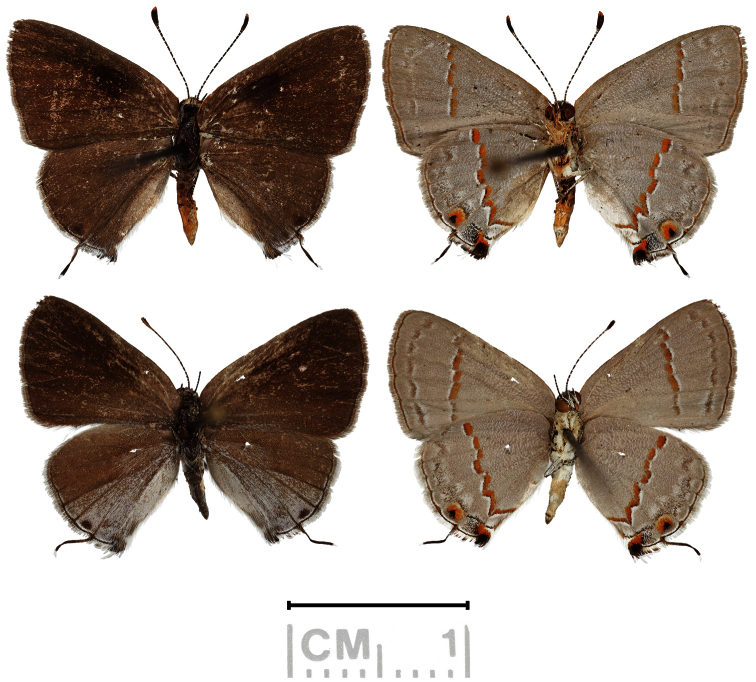
Male holotype (top) and female paratype of *Ministrymon janevicroy*. Eye color in the male appears to have darkened more post mortem than that of the female.

Adults of *Ministrymon janevicroy* have olive green eyes in nature while those of *Ministrymon azia* have dark brown/black eyes (Fig. 1). The 30 images of adults in nature with a variegated “pebbly-textured” basal hindwing have olive green eyes, and the 44 images of those with a smooth-textured gray basal hindwing have dark brown/black eyes. In the museum study series, all *Ministrymon azia* adults had dark brown/black eyes while 9.5% of *Ministrymon janevicroy* adults had eyes as dark as those of *Ministrymon azia* (data in a supplementary file). The remaining adults of *Ministrymon janevicroy* had lighter eyes, ranging from yellow-brown to brown (this variation is shown in Fig. 3). It would appear that eye color darkens a variable amount post mortem in *Ministrymon janevicroy*. A survey of eye color in other *Ministrymon* species is presented in the discussion.

The wing venation of male and female *Ministrymon janevicroy* is illustrated (Fig. 8). In *Ministrymon janevicroy* forewing vein M_2_ arises closer to M_1_ than to M_3_ in both sexes, but is otherwise typical of the Eumaeini (Eliot 1973). Males of *Ministrymon janevicroy* have a scent patch at the distal end of the forewing discal cell in which the tan androconia are partially or wholly (in some individuals) covered by dark brown wing scales (Fig. 9). This scent patch structure is the same as that in *Ministrymon azia*. There is no evident sexual dimorphism in size (♂ mean forewing length = 9.1 mm, s=0.62, N=10, ♀ mean forewing length = 9.1 mm, s=0.33, N=4, data in supplementary file).

#### Distribution, habitat, and phenology.

**Figure 4.**
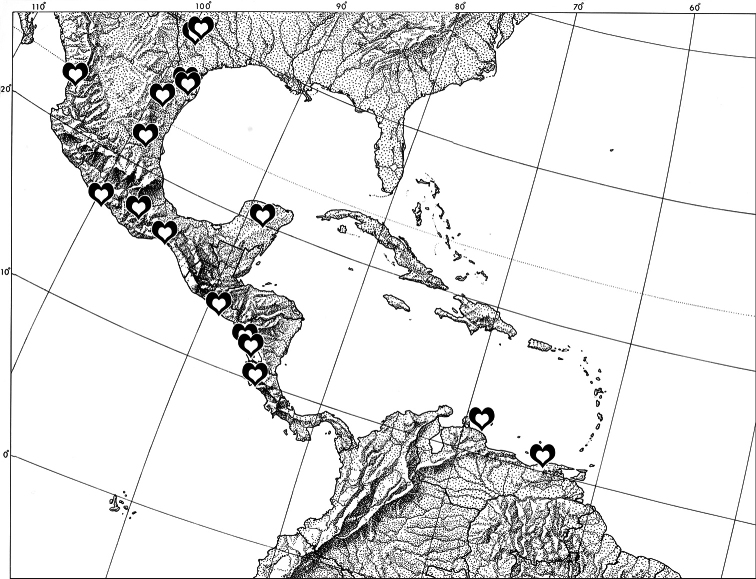
Distribution of *Ministrymon janevicroy* (hearts) based on museum specimens.

*Ministrymon janevicroy* occurs from southern Texas (there is also an image of an individual of this species from Big Bend National Park in western Texas, cf. supplementary information) to Guanacaste Province, Costa Rica and in South America on the islands of Curaçao and Margarita (Venezuela) (Fig. 4). It is a relatively common species in Central America, where it is as well represented in museum collections as *Ministrymon azia*. *Ministrymon janevicroy* appears to be absent from the Antilles (including Florida and the Lesser Antilles) and from South America, except for Curaçao and Venezuela’s Isla Margarita. It may also occur on Aruba, where *Ministrymon azia* was recorded (Miller et al. 2003), but we have not seen specimens. *Ministrymon janevicroy* inhabits dry deciduous forest and scrub. It and *Ministrymon azia* occur at the same localities. For example, both have been collected at the type locality for *Ministrymon janevicroy* (Santa Ana Wildlife Refuge) in Hidalgo County, and both were photographed on the same day at the same locality (Rio Blanco Canyon) near Orizaba, Veracruz, Mexico. In Texas, adults of *Ministrymon janevicroy* have been found from January to August. Elsewhere, there is no evidence for seasonality.

**Figure 5.**
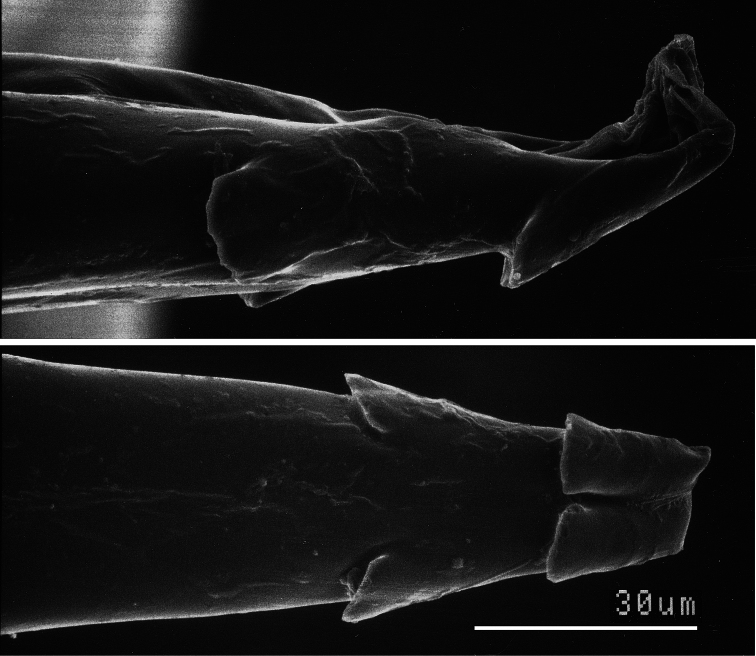
SEM of *Ministrymon azia* penis tip showing small erect teeth in lateral (top) and ventral aspect.

**Figure 6.**
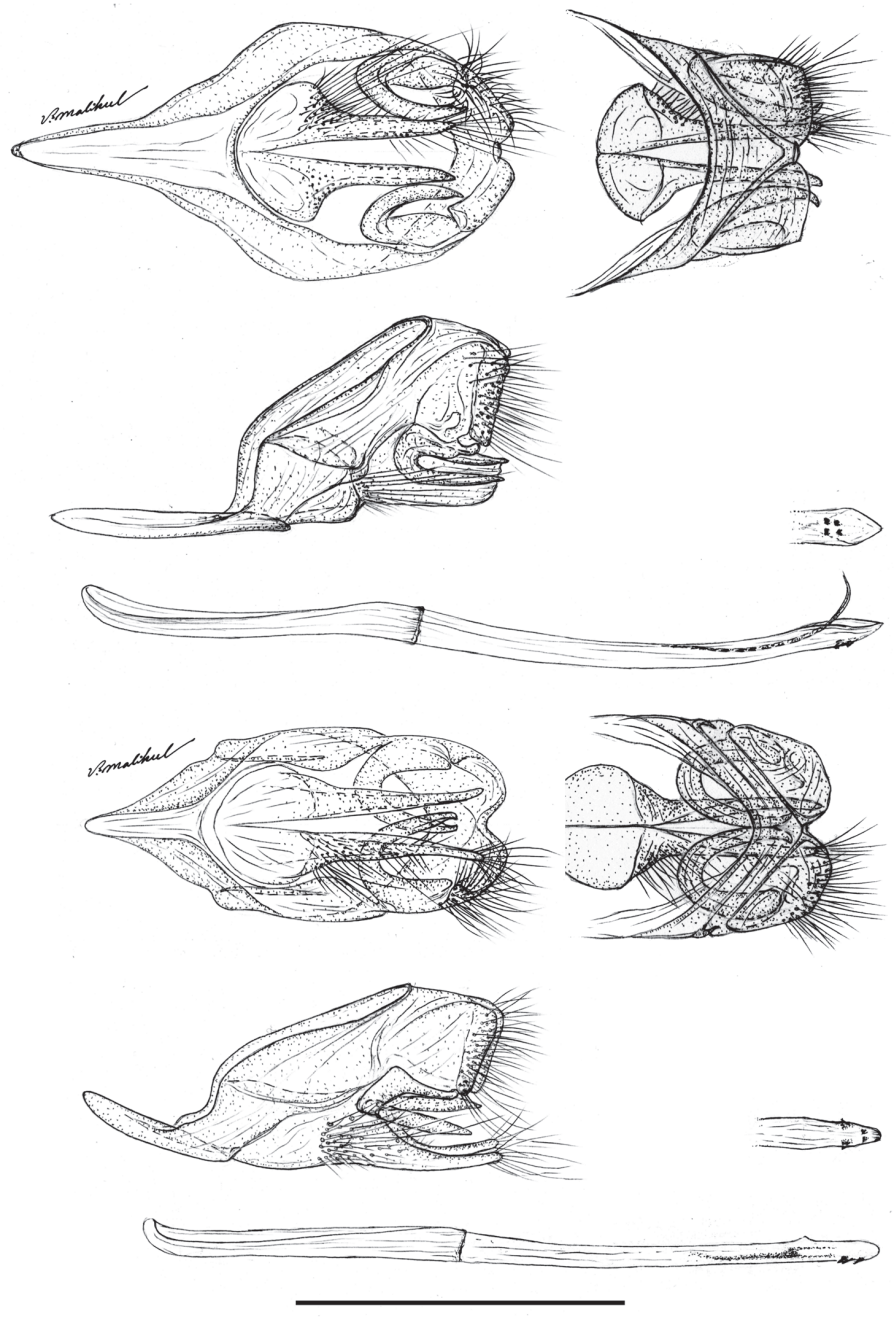
Male genitalia of *Ministrymon janevicroy* (top) and *Ministrymon azia*, posterior of butterfly at right, both from Yucatan, Mexico. Ventral aspect with penis removed (top left), lateral aspect with penis removed (left middle), lateral aspect of penis (bottom), penis tip in ventral aspect (right middle), and dorsal aspect of tegumen (top right). Scale 1 mm.

**Figure 7.**
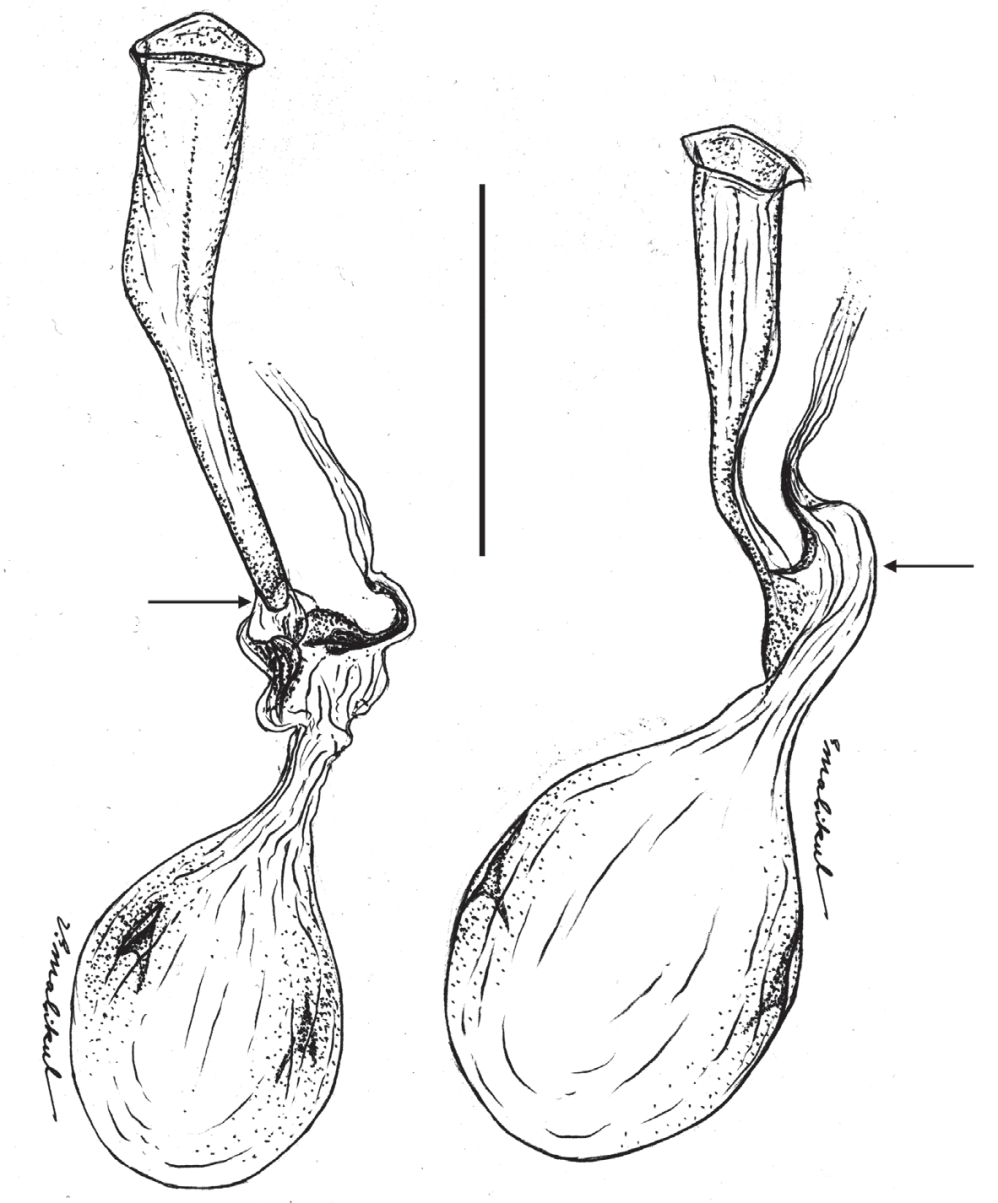
Bursa copulatrix of the female genitalia of *Ministrymon janevicroy* (left, Venezuela) and *Ministrymon azia* (Mexico) in dorso-lateral aspect, posterior of butterfly at top. Arrow on left points to the membranous “neck” of the anterior ductus bursae. Arrow on right points to the well-formed posterior pouch from which the ductus seminalis arises. Scale 1 mm.

**Figure 8.**
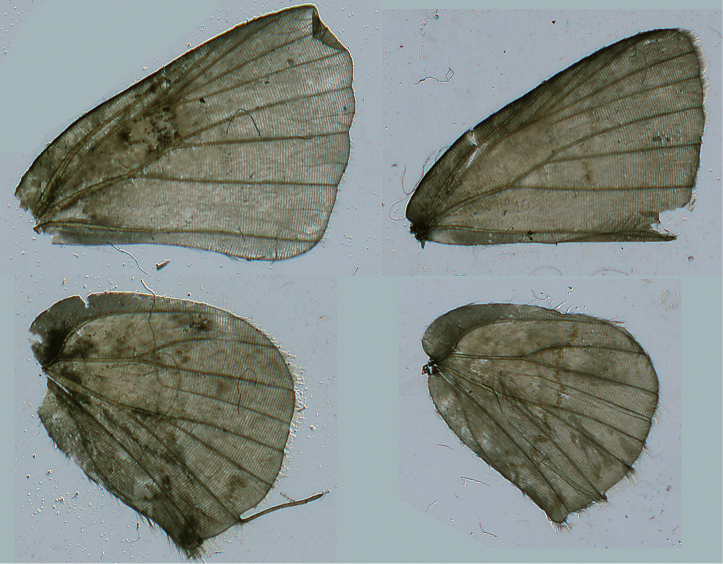
Male (left, Yucatan, Mexico) and female (Santa Tecla, El Salvador) venation of *Ministrymon janevicroy*.

**Figure 9.**
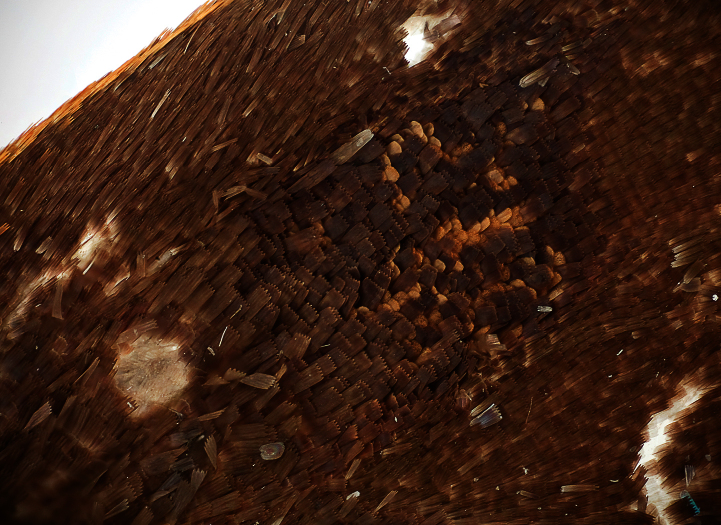
Male dorsal forewing scent patch showing dark brown wing scales covering tan-colored androconia.

## Discussion

**Generic Placement and Identification**. *Ministrymon janevicroy* is placed in *Ministrymon* because it possesses small erect teeth on the ventral surface of the penis near the distal end (Fig. 6). This synapomorphy for *Ministrymon* was proposed by Clench (1961) and has not been reported in other eumaeine genera. Other characters accord with this placement (Robbins unpubl.). Forewing vein M_2_ arises closer to M_1_ than to M_3_ in the male (Fig. 8) and the corpus bursae of the female genitalia is posteriorly constricted (Fig. 7). These traits are widespread (but not universal) in the *Tmolus* Section of Eumaeini, to which *Ministrymon* belongs (Robbins 2004a). A male dorsal scent patch is situated at the distal end of forewing discal cell and is partially covered by dark brown wing scales (Fig. 9). Within the *Tmolus* Section, this type of scent patch occurs in all *Ministrymon*, all *Tmolus*, and some *Nicolaea* K. Johnson.

The hypothesis that *Ministrymon janevicroy* is reproductively isolated from the sympatric *Ministrymon azia* is well-supported. The male and female genitalic differences between the two are distinct and distinguishing (Figs 6–7). The variegated “pebbly-textured” ground color appearance of the basal part of the ventral hindwing (Fig. 2) is also distinguishing. The eye color difference is distinct and distinguishing in live individuals (Fig. 1) and in the majority of museum specimens. *Ministrymon janevicroy* is unrecorded from tropical wet lowland forest (>200 cm annual precipitation, Holdridge 1967) while *Ministrymon azia* occurs in both wet and dry habitats. *Ministrymon janevicroy* occurs from Texas to Costa Rica and on Curaçao and Isla Margarita. In contrast, *Ministrymon azia* occurs commonly in wet and dry habitats from Texas and Florida to southern Brazil and Argentina. In sum, the two species differ morphologically and biologically in many respects, supporting a hypothesis of reproductive isolation between *Ministrymon janevicroy* and *Ministrymon azia*.

The substantive differences in the genitalic structures of *Ministrymon azia* and *Ministrymon janevicroy* (Figs 6–7) could be interpreted to mean that these two taxa are not closely related within *Ministrymon*. However, these species are sympatric in the same habitats, have very similar wing patterns, and the same androconial structures. If reproductive isolation between these species evolved by sexual selection acting on the genitalia (e.g., Eberhard 1985, Arnqvist 1998, Hosken and Stockley 2004), then the genitalic differences observed could be a consequence of this evolutionary process, and is not an indication of a lack of relationship.

In the diagnosis and previous paragraphs, we distinguished *Ministrymon janevicroy* from *Ministrymon azia* because both share a similar ventral wing pattern. If *Ministrymon janevicroy* were more closely related to another described *Ministrymon* species, its ventral wing pattern would distinguish it immediately from that species.

**Eye Color**. The “hairiness” of adult eyes has been widely used in lycaenid taxonomy for more than a century (cf. Eliot 1973 and included references), but adult eye-color has not been used traditionally (e.g., Godman and Salvin 1887-1901, Scudder 1889, Draudt 1919-1920, Eliot 1973). More recently, Glassberg (2001, 2005) used eye color in live individuals to differentiate the lycaenid *Cyanophrys goodsoni* (Clench) from other *Cyanophrys* species and to distinguish between the lycaenids *Strymon bazochii* (Godart) and *Strymon cestri* (Reakirt). *Ministrymon janevicroy* was originally discovered in Texas and Mexico (Glassberg 2005) because its adults have olive green eyes in nature instead of the dark brown/black eyes of *Ministrymon azia*. Eye color in *Ministrymon janevicroy* appears to darken after death, but most museum specimens of *Ministrymon janevicroy* have lighter eyes than those of *Ministrymon azia*.

To provide context for the eye color difference between *Ministrymon azia* and *Ministrymon janevicroy*, we surveyed other *Ministrymon* species by recording eye color in museum specimens and in images of live adults. *Ministrymon zilda* (Hewitson) has a deep black eye color (an apparent autapomorphy), both in museum specimens and in live individuals. *Ministrymon cleon* (Fabricius) (cf. Duarte and Robbins 2010 for a note on identification of this name) has the same dark brown/black eyes as *Ministrymon azia* in museum specimens and in one image of a live adult. Museum specimens of all other described *Ministrymon* species are variable with most having lighter-colored eyes than those of *Ministrymon azia*—similar in color to those of *Ministrymon janevicroy*—and with a minority of individuals in each species having the dark brown/black-colored eyes of *Ministrymon azia*. Of these, we have examined images of live adults for nine species, all of which have olive green eyes.

This survey of *Ministrymon* adult eye colors leads to three conclusions. First, although similarity in wing pattern suggests that *Ministrymon azia* is the phylogenetic sister of *Ministrymon janevicroy*, adult eye color suggests that *Ministrymon azia* might be the phylogenetic sister of *Ministrymon cleon*. Clearly, a phylogenetic analysis of the genus is needed. Second, adult eye color is a useful taxonomic character in the field, but its use in the museum is more limited. In this case, all museum specimens in the “*Ministrymon azia*” complex with yellow-brown to brown eyes are *Ministrymon janevicroy*, but the converse is untrue. Third, the biological significance of adult eye color is yet unknown. There is no evident correlation with gender, habitat, or other biological traits.

**Variation**. While there was no discernible geographic variation in the morphology of *Ministrymon janevicroy*, three morphological aspects vary in single populations. First, when Hewitson (1873) described *Ministrymon azia*, he noted that the discal spot (= scent patch) was “indistinctly marked” in one male and was more conspicuous in another male. The same variation occurs in *Ministrymon janevicroy*. It is caused by variation in the color of the dark brown scales covering the scent patch (Fig. 9), not by variation in the presence of androconia. Second, there may be four or five small erect teeth on the penis, and in one dissection, there was an indication of yet another tooth. Third, as previously noted, eye color varies in museum specimens depending upon postmortem darkening, but this variation is not reflected in live individuals, so far as we are aware.

**Biogeography**. A number of Central American eumaeine species occur in deciduous dry forest from the southern United States (Texas) or Mexico to Costa Rica (Guanacaste), but are unrecorded further south and east in Panama and northern Colombia. This species list includes *Arawacus sito* (Boisduval), *Cyanophrys goodsoni* (Clench), *Cyanophrys miserabilis* (Clench), *Michaelus hecate* (Godman & Salvin), *Ministrymon clytie* (W.H. Edwards), *Rekoa zebina* (Hewitson), *Strymon alea* (Godman & Salvin), *Strymon bebrycia* (Hewitson), and *Ziegleria hoffmani* (K. Johnson). *Ministrymon janevicroy* is now added to this list. Of these, only *Strymon alea* (Isla Margarita) and *Ministrymon janevicroy* are also recorded on islands just off the north coast of South America, but not from the dry continental forests of northern Venezuela and northern Colombia. It is possible that remnant populations of species that were once more widespread persist only on these islands, but alternately, the mainland Guajira peninsula of Colombia/Venezuela is yet poorly documented.

There is one female in the *Ministrymon azia* species group in the USNM from the Brazilian state of Minas Gerais that has the olive green eye color and variegated “pebbly-textured” wing pattern of *Ministrymon janevicroy*, but a different postmedian line on the ventral surface of the hindwing. Additionally, two males of the same species in MC are genitalically distinct from *Ministrymon janevicroy* (genitalic images sent to us by A. Moser). This species is a potential phylogenetic sister species of *Ministrymon janevicroy*. We are collaborating with Moser to find more specimens with the intention of describing it.

**Nomenclature**. Seven names have been applied to the species now called *Ministrymon azia* (Robbins 2004a), and it is the purpose of this section to explain why these names do not apply to *Ministrymon janevicroy*. The name *Thecla guacanagari* Wallengren, 1860 (Ecuador), which has a ventral wing pattern that appears to be transitional between that of *Thecla azia* and *Thecla brocela*, was suppressed by ICZN Opinion 2144 (ICZN 2006). We examined the lectotype of *Thecla azia* Hewitson, 1873 (Mexico) in the BMNH and received images of its male genitalia (courtesy R.I. Vane-Wright and B. Huertas). It is the basis for our identification of this name. We examined the holotype of *Thecla nipona* Hewitson, 1877 (Brazil). It has the smooth-textured gray appearance on the basal half of the ventral wing surface. Also, *Ministrymon janevicroy* does not occur in Brazil. We examined the holotype of *Thecla brocela* Dyar, 1913 (USNM). Its ventral wing pattern is typical of those populations of *Ministrymon azia* that occur west of the Andes from southern Ecuador to Chile. The genitalia, gray smooth-textured appearance on the basal half of the ventral wing surface, and uniform dark brown/black eyes are the same as those of *Ministrymon azia* from elsewhere. We examined an image of the holotype of *Ministrymon quebradivaga* K. Johnson & L.D. Miller, 1991 (Chile). As with *Ministrymon brocela*, its wing pattern is typical of those populations of *Ministrymon azia* that occur west of the Andes from southern Ecuador to Chile. The genitalia illustrated in the original description are those of *Ministrymon azia*, not of *Ministrymon janevicroy*. We know *Ministrymon hernandezi* Schwartz & K. Johnson, 1992 (Cuba) from the original publication. The male genitalia illustrations are stylized renderings, but the placement of the teeth on the ventral penis tip and the shape of the cornuti place this name in the synonymy with *Ministrymon azia*. Further, *Ministrymon janevicroy* does not occur in the Antilles. Finally, we accord nomenclatural priority to *Angulopis hernandezi* K. Johnson & Kroenlein, 1993 (a male holotype) over *Electrostrymon grumus* K. Johnson & Kroenlein, 1993 (a female holotype), **new synonym**, and remove the latter name from the synonymy of *Ministrymon azia*. Robbins (2004a) had erroneously listed *grumus* as a synonym of *hernandezi* Schwartz & K. Johnson rather than as a synonym of *hernandezi* K. Johnson & Kroenlein. The name *Angulopis hernandezi* is currently placed in genus *Ziegleria* K. Johnson (Robbins 2004a, Duarte and Robbins 2010).

**Faunal documentation**. The Eumaeini fauna of the United States is well-documented, and most species described in the past 75 years have arguably been cryptic species that had been overlooked because of wing pattern similarity with known species. Specific examples are *Satyrium caryaevorus* (McDunnough), *Satyrium kingi* (Klots & Clench), *Callophrys hesseli* (Rawson & Ziegler), and *Strymon solitario* Grishin & Durden. To this list, we add *Ministrymon janevicroy*. In sharp contrast, slightly more than 20% of the Central and South American eumaeine fauna is undescribed (Robbins 2004b), but the vast majority of these undescribed species are exceedingly rare in museum collections, unlike *Ministrymon janevicroy*. Assessing variation of these rare species remains an obstacle to documentation.

**Biology and updated taxonomy**. The purpose of this section is to assess previously published biological information about *Ministrymon azia* in the context of the updated taxonomy. As noted in the introduction to this paper, *Ministrymon azia* occurs from the United States (Texas and Florida) to Chile in virtually all lowland habitats, ranging from desert in coastal Peru and Chile to rainforest in the Amazon Basin. The information in this paper is consistent with this statement, and we note that there are specimens of *Ministrymon azia* in the USNM from Hidalgo, Cameron, and Edwards County in Texas. *Ministrymon janevicroy* is restricted to dry deciduous forest and scrub, and its range is a subset of that of *Ministrymon azia*. These species appear to be sympatric wherever *Ministrymon janevicroy* occurs.

*Ministrymon azia* was the most common lycaenid migrating through Portachuelo Pass in northern Venezuela (Beebe 1951), and it was one of the species being dispersed by dry season trade winds on the Pacific slope of Panama (Robbins and Small 1981). There are 34 vouchers of *Ministrymon azia* and none of *Ministrymon janevicroy* in the USNM that were caught migrating through Portachuelo Pass. There are 12 vouchers of *Ministrymon azia* and none of *Ministrymon janevicroy* in the USNM that were being dispersed by dry season trade winds at the Cerro Campana ridge in Panama. The records of migration and dispersal would appear to refer to *Ministrymon azia*, not to *Ministrymon janevicroy*.

Published larval food plant records (all Fabaceae, one exception) for *Ministrymon azia* from areas where *Ministrymon janevicroy* does not occur are the plant genera *Acacia* Willd. in the United States (Florida) and Chile, *Mimosa* L. in Cuba, and Trinidad, and *Leucaena* Benth. in the United States (Florida) (Boscoe 1982, Cock 1985, Fernández and Rodríguez 1997, Vargas and Parra 2009, Glassberg 2012, images of the immatures of *Ministrymon azia* on *Leucaena* available from Chin-Lee, http://bugguide.net/node/view/133700, accessed April 22, 2013). Miller et al. (1997) wrote that caterpillars of *Ministrymon azia* in Florida will eat *Schinus terebinthifolius* Raddi (Anacardiaceae), but this anomalous record needs confirmation.

Reared museum specimens that we have seen have all been *Ministrymon azia*. We examined individuals of *Ministrymon azia* in the USNM that were reared from *Leucaena* in Florida (2♂, 2♀) and from *Mimosa* in Guerrero and Veracruz, Mexico (1♂, 1♀) and in Trinidad (2♀). In DZUP, there are reared specimens from *Mimosa* in Pernambuco and Rio Grande do Sul, Brazil (2♂). Finally, we identified an adult female that was reared from *Prosopis* L. in Tarapacá, Chile, but deposition of this specimen is unknown.

Other caterpillar food plant records are currently ambiguous and may refer to *Ministrymon azia* or to *Ministrymon janevicroy*. Harley et al. (1995) reared “*Ministrymon azia*” in Mexico and Venezuela from *Mimosa*, but the deposition of the vouchers is not known. In TAMU, there are reared adults from *Leucaena* (3♂, 3♀) and from *Mimosa* (4♂, 3♀) in the United States (Texas). In UCR, there are two reared individuals from *Leucaena* in Sonora, Mexico. These reared individuals need to be re-examined to determine their specific identify.

## Supplementary Material

XML Treatment for Ministrymon janevicroy

## Appendix 1

List of genitalic dissections. (doi: 10.3897/zookeys.305.5081.app1) File format: Adobe PDF file (pdf).

## Appendix 2

Images of live butterflies examined. (doi: 10.3897/zookeys.305.5081.app2) File format: Adobe PDF file (pdf).

## Appendix 3

Data on forewing length and frequency of eye-color. (doi: 10.3897/zookeys.305.5081.app3) File format: Adobe PDF file (pdf).
