# Supplementary material for: A butterfly with olive green eyes discovered in the United States and the Neotropics (Lepidoptera, Lycaenidae, Eumaeini)
Source: Zookeys. 2013 May 28;(305):1–20. doi: 10.3897/zookeys.305.5081 (PMC3689090; doi:10.3897/zookeys.305.5081)
Supplement: Supplementary file 2 — List of genitalic dissections. (doi: 10.3897/zookeys.305.5081.app1) File format: Adobe PDF file (pdf). [file ZooKeys-305-001-s001.pdf]

MINISTRYMON genitalia, *azia* and *janevicroy* project

2009:78 ♂	Ministrymon	janevicroy	10 mi. W Victoria, Mexico	USNM
2009:79 ♂	Ministrymon	janevicroy (genitalia illustrated)	Chicken Itza, Mexico	USNM
2009:80 ♂	Ministrymon	janevicroy	20 mi N Juchitan, Mexico	USNM
2009:95 ♂	Ministrymon	janevicroy	Santa Ana Ref., Hidalgo, TX	USNM
WF1928♂	Ministrymon	janevicroy (Field dissection not found)		
			Concan, TX	USNM
2009:94 ♂	Ministrymon	janevicroy	Guanacaste, Costa Rica	USNM
2013:09 ♂	Ministrymon	janevicroy	Curacao	FSMC
2013:10 ♂	Ministrymon	janevicroy (holotype)	Santa Ana Ref., Hidalgo, TX	USNM
2009:81 ♀	Ministrymon	janevicroy	Managua, Nicaragua	USNM
2009:82 ♀	Ministrymon	janevicroy	Kerrville, TX	USNM
2009:83 ♀	Ministrymon	janevicroy (genitalia illustrated)	Margarita, Venezuela	USNM
2009:84 ♀	Ministrymon	janevicroy	Sullivan City, TX	USNM
2009:85 ♀	Ministrymon	janevicroy	10 mi S Victoria	USNM
2013:02 ♀	Ministrymon	janevicroy	Margarita, Venezuela	USNM
2002: 46♀	Ministrymon	new species	MG, Brazil	USNM
	(Female genitalia same as M. janevicroy, but male, vide Moser, is very different)			
2009:86 ♂	Ministrymon	azia (genitalia illustrated)	Chicken Itza, Mexico	USNM
2009:96 ♂	Ministrymon	azia (Sullivan dissection)	Cali, Colombia	USNM
L529 ♂	Ministrymon	azia (Nicolay dissection)	Salta, Argentina	USNM
1981: 4 ♂	Ministrymon	azia	Villavicencio, Colombia	USNM
2009:97♂	Ministrymon	azia (Nicolay dissection)	Loja, Ecuador	USNM
2009:98♂	Ministrymon	azia (Nicolay dissection)	Loja, Ecuador	USNM
2009:89 ♂	Ministrymon	azia	Valle, Colombia	USNM
2009:90 ♂	Ministrymon	azia	Santa Ana Ref, Hidalgo, TX	USNM
2009:91 ♂	Ministrymon	azia	Brownsville, TX	USNM
2009:92 ♂	Ministrymon	azia	Brownsville, TX	USNM
WF5179♂	Ministrymon	azia (Field dissection, holotype brocela)		
			Cotahusi, Peru	USNM
2009:87 ♀	Ministrymon	azia	Cerro Campana, Panama	USNM
2009:88 ♀	Ministrymon	azia (genitalia illustrated)	Jalapa, Mexico	USNM
2009:99 ♀	Ministrymon	azia	Loja, Ecuador	USNM
L548 ♀	Ministrymon	azia (dissection not found)	Cerro Campana	USNM
2009:93 ♀	Ministrymon	azia	Valle, Colombia	USNM
2013:01 ♀	Ministrymon	azia	Paint Creek, Edwards Co, TX	USNM
