# Supplementary material for: A butterfly with olive green eyes discovered in the United States and the Neotropics (Lepidoptera, Lycaenidae, Eumaeini)
Source: Zookeys. 2013 May 28;(305):1–20. doi: 10.3897/zookeys.305.5081 (PMC3689090; doi:10.3897/zookeys.305.5081)
Supplement: Supplementary file 3 — Images of live butterflies examined. (doi: 10.3897/zookeys.305.5081.app2) File format: Adobe PDF file (pdf). [file ZooKeys-305-001-s002.pdf]

Ministrymon janevicroy. 30 images of live butterflies examined.
(all, except for those in A Swift Guide to Butterflies of North America, are listed as Ministrymon azia, Gray Ministreak)

Texas. 19 images.
2006 Jan 14. National Butterfly Center, Hidalgo Co. Gil Quintanilla & Dave Hanson
http://www.naba.org/chapters/nabast/gmini.html
2013 Mar 23. Falcon Heights, Starr Co. Berry Nall.
Private collection.
2008 Apr 19. National Butterfly Center, Hidalgo Co. Jeffrey Glassberg
Swift Guide to Butterflies of North America, pg. 117, inset photo
2012 Apr 28. Falcon Heights, Starr Co. Berry Nall.
http://leps.thenalls.net/speciesnum.php?lep=101
2009 May 1. Falcon Heights, Starr Co. Berry Nall.
http://leps.thenalls.net/speciesnum.php?lep=101
2009 May 1. Falcon Heights, Starr Co. (second individual). Berry Nall.
http://leps.thenalls.net/speciesnum.php?lep=101
2009 May 2. Falcon Heights, Starr Co. Berry Nall.
http://leps.thenalls.net/speciesnum.php?lep=101
2009 May 6. Falcon Heights, Starr Co. Berry Nall.
http://leps.thenalls.net/speciesnum.php?lep=101
2009 May 8. Falcon Heights, Starr Co. Berry Nall.
http://leps.thenalls.net/speciesnum.php?lep=101
2011 May 7. Falcon Heights, Starr Co. Berry Nall.
http://leps.thenalls.net/speciesnum.php?lep=101
2008 May 10. Falcon Heights, Starr Co. Berry Nall.
http://leps.thenalls.net/speciesnum.php?lep=101
2009 May 11. Falcon Heights, Starr Co. Berry Nall.
http://leps.thenalls.net/speciesnum.php?lep=101
2000 May 20. Oak Creek Canyon, Big Bend National Park, Brewster Co. Ro Wauer
Private collection.
2009 June 14. Bentson World Birding Center, Hidalgo Co. Gil Quintanilla
http://www.naba.org/sightings/archives/jun2009/grayms.htm
2011 June 17. Sabal Palm Sanctuary, Cameron Co. Lee Hoy.
http://www.flickr.com/photos/leehoy/5851436716/
2012 June 20. Dugout Wells, Big Bend National Park, Brewster Co. Lee Hoy.
http://www.flickr.com/photos/leehoy/7640826206/
2009 June 27. Falcon Heights, Starr Co. Berry Nall.
http://leps.thenalls.net/speciesnum.php?lep=101
2009 July 1. Falcon Heights, Starr Co. Berry Nall.
Private collection.
2007 Aug 22. Bentsen State Park, Hidalgo Co. Joshua S. Rose
http://bugguide.net/node/view/139098/bgpage

Mexico. 10 images.
2001 Apr 28, Colonia Francisco Barrios, Veracruz. Jeffrey Glassberg
Swift Guide to the Butterflies of Mexico and Central America, pg. 46.
2008 June 9, Rio Blanco Canyon, near Orizaba, Veracruz. Jeffrey Glassberg
Swift Guide to Butterflies of North America, pg. 117 main photo
2005 Aug. 9. Near Yecora, Sonora. Kim Davis & Mike Stangeland.
http://kimandmikeontheroad.com/gray_ministreak.htm
2005 Aug. 9. Ruta 16, Sonora. Kim Davis & Mike Stangeland.

http://kimandmikeontheroad.com/gray_ministreak.htm
2008 Sept 2. Yecora, Sonora. Kurt Radamaker.
http://mexicobirding.com/gallery2/main.php?g2_itemId=390
1998. Sept. 5. Chipinque Park, Monterrey, Nuevo Leon. Jeffrey Glassberg.
Glassberg J (2005) Butterfly Eye-dentification. American Butterflies 13(2): 41.
2009 Sept 10, Barranca Honda-Rio Yautepec, Morelos. Luc Legal, Jerome Albre and Oscar Dorado
http://www.butterfliesofamerica.com/L/ministrymon_azia_live1.htm
2004 Nov 5. Los Troncones, Tamaulipas. Will Carter
http://neotropicalbutterflies.com/Site%20Revision/Pages/HairstreakPages/Tmolus_Section/Tmolus_Pages/Ministrymon_azia.html
2004 Nov 7. Los Troncones, Tamaulipas. Richard Lehman
http://neotropicalbutterflies.com/Site%20Revision/Pages/HairstreakPages/Tmolus_Section/Tmolus_Pages/Ministrymon_azia.html
2004 Nov. 18. Hotel Taninul, San Luis Potosi.
http://kimandmikeontheroad.com/gray_ministreak.htm

Venezuela. 1 image.
March 15, 1988. Margarita Island. Jeffrey Glassberg
Glassberg J (2005) Butterfly Eye-dentification. American Butterflies 13(2): 41.

Ministrymon azia. 43 images of live butterflies examined.
Texas. 4 images.
2009 May. Bentsen-Rio Grande Valley State Park, Hidalgo Co. Mary Beth Stowe
http://www.miriameaglemon.com/photo_gallery/Butterflies/LRGV/Hairstreaks.html
2011 May 4. National Butterfly Center, Hidalgo Co. Jeffrey Glassberg
private collection.
2012 Dec. 17. National Butterfly Center, Hidalgo Co. Dave Hanson.
http://sightings.naba.org/sightings/archive/2012/12
2004 Dec. 19. National Butterfly Center, Hidalgo Co. Dave Hanson
Glassberg J (2005) Butterfly Eye-dentification. American Butterflies 13(2): 41.

Mexico. 10 images.
2008 June 2. Lake Catemaco, Veracruz. Kim Davis & Mike Stangeland.
http://www.butterfliesofamerica.com/L/ministrymon_azia_live2.htm
2008 June 3. Lake Catemaco, Veracruz. Kim Davis & Mike Stangeland.
http://www.butterfliesofamerica.com/L/ministrymon_azia_live2.htm
2008 June 3. Lake Catemaco, Veracruz. Kim Davis & Mike Stangeland.
http://www.butterfliesofamerica.com/L/ministrymon_azia_live2.htm
2008 June 5. Lake Catemaco, Veracruz. Kim Davis & Mike Stangeland.
http://www.butterfliesofamerica.com/L/ministrymon_azia_live2.htm
2008 June 9. Rio Blanco Canyon, nr. Orizaba, Veracruz. Jeffrey Glassberg
Private collection.
2008 June 11. Huatusco, Veracruz. Jeffrey Glassberg
Private collection
2009 July 30. Chavarillo, Veracruz. Jeffrey Glassberg
Swift Guide to Butterflies of North America, pg. 117, main photo
2007 Aug 20. Metates, Oaxaca. Jeffrey Glassberg
Swift Guide to Butterflies of North America, pg. 117, inset photo
2007 Aug. 20-Sept. 15. Mpio. Santa Maria Atzompa, Oaxaca. Angel G. Vasquez
http://www.butterfliesofamerica.com/L/ministrymon_azia_live1.htm
2004 Nov. 22. Hotel Taninul, San Luis Potosi. Kim Davis & Mike Stangeland.
http://kimandmikeontheroad.com/gray_ministreak.htm

Ecuador. 3 images.

2011 Mar 29. Panamerican Hwy, San Antonio Rd at 4,154 ft, Catacocha, Loja Prov. “gmanager999”
http://www.flickr.com/photos/gmanager999/8088939183/
date unknown. Reserva Geobotánica Pululahua   Prov. Pichincha. Photographer unknown.
http://www.pululahuahostal.com/html/butterflies_of__pululahua.html
2009 Nov 20. West slope near Vilcabamba, Rio Yambala. Kim Garwood.
Private collection

Trinidad. 1 image.
2012 Feb. 5. Rousillac. Kris Sookdeo.
http://gallery.ttnaturelink.com/Insects/Butterflies-of-Trinidad-Tobago/Lycaenidae/Theclinae/LLY-Ministrymon-azia-Butterflies-of-Trinidad-and-Tobago-Wildlife-Nature

Brazil. 2 images.
2010 Feb 9. Reserva Ecologia de Guapiacu, Rio de Janeiro. Kim Garwood
Private collection
2011 Oct. 4. Cristalino Jungle Lodge, Mato Grosso. Rich Hoyer.
http://www.flickr.com/photos/birdernaturalist/8129951889/

Paraguay. 1 image.
unknown date. Unknown location within Paraguay. Unknown photographer.
http://www.pybio.org/en/1183/lycaenidae/

Florida. 15 images.
2005 June 5. Bauer Hammock, Homestead, Miami-Dade Co. Linda Cooper
http://www.naba.org/sightings/archives/june2005/graymini.htm
2012 July 4. Hampton Pines Park, Broward Co. Larry Milligan.
http://www.flickr.com/photos/larryfl/7581300628/
2012 July 17. South Florida. Bart Jones.
http://www.flickr.com/photos/bjones7777/7601334686/
2003 July 6. Miami-Dade Greenway, Miami-Dade Co. Dave Powell.
http://www.vireos.com/grayministreak.html
2005 July 28. Fort DeSoto, Pinellas Co. “Pondhawk”
http://www.flickr.com/photos/38686613@N08/4731417771/
2007 July 29. Miami, Miami-Dade Co. Alan Chin-Lee
http://bugguide.net/node/view/132183
2004 Aug. 1. Castellow Hammock, Miami-Dade Co. Linda Cooper.
http://www.naba.org/sightings/archives/august2004/grayministreak.htm
2007 Aug 3. Homestead, Miami-Dade Co. Alan Chin-Lee.
http://bugguide.net/node/view/133700
2008 Aug. 4. Castello Hammock, Miami-Dade Co. Alana Edwards.
Private collection.
2003 Aug. 9. Homestead, Miami-Dade Co. Jeffrey Pippen.
http://people.duke.edu/~jspippen/butterflies/grayministreak.htm
2008 Aug 23. Castellow Hammock, Miami-Dade Co. Barbara Woodmansee
http://ceraunus.zenfolio.com/p585166066/h13cd5922#h13cd5922
2001 Aug 27. Homestead, Miami-Dade Co. Jeffrey Glassberg
Glassberg J (2005) Butterfly Eye-dentification. American Butterflies 13(2): 41.
2001 Sept. 3. Homestead, Miami-Dade Co. Dave Powell.
http://www.vireos.com/grayministreak.html
2003 Sept. 18. Miami-Dade Co. Jeffrey Pippen.
http://people.duke.edu/~jspippen/butterflies/grayministreak.htm
2011 Dec 21. Miami, Miami-Dade Co. Barbara Woodmansee
http://ceraunus.zenfolio.com/p585166066/h3DD0BCBD#h3dd0bcbd

Jamaica. 1 image.
2006 March 16. Saint Mary. Paul Thomas.
http://www.flickr.com/photos/paulthomasdigital/113440722/

Puerto Rico. 3 images.
2010 July 17. Unknown location on Puerto Rico. Miguel Angel.
http://www.flickr.com/photos/miguel1pr/6355823831/
2011 Sept. 5. Unknown location on Puerto Rico. Miguel Angel.
http://www.flickr.com/photos/miguel1pr/6165122208/
2008 Nov. 8. Guanica. Alana Edwards.
Private collection.

Dominican Republic. 1 image.
2002 Nov. 6. Barahona. Jeffrey Glassberg.
Glassberg J (2005) Butterfly Eye-dentification. American Butterflies 13(2): 41.

Saint Martin. 3 images.
2013 Jan. 11. Unknown location on Saint Martin. Marc AuMarc.
http://www.flickr.com/photos/theactionitems/8371989890/
2013 Jan. 5. Unknown location on Saint Martin. Marc AuMarc.
http://www.flickr.com/photos/theactionitems/8351336882/
2012 Aug. 7. Unknown location on Saint Martin. Marc AuMarc.
http://www.flickr.com/photos/theactionitems/7740583706/
