# Supplementary material for: A butterfly with olive green eyes discovered in the United States and the Neotropics (Lepidoptera, Lycaenidae, Eumaeini)
Source: Zookeys. 2013 May 28;(305):1–20. doi: 10.3897/zookeys.305.5081 (PMC3689090; doi:10.3897/zookeys.305.5081)
Supplement: Supplementary file 4 — Data on forewing length and frequency of eye-color. (doi: 10.3897/zookeys.305.5081.app3) File format: Adobe PDF file (pdf). [file ZooKeys-305-001-s003.pdf]

Male *M. janevicroy* FW lengths, mean=9.1mm, s=0.62, N=10

9.2
10.3
8.7
8.4
9.6
9.2
8.5
8.4
8.8
9.5

Female *M. janevicroy* FW lengths, mean=9.1mm, s=0.33, N=4

9.3
9.0
9.3
8.6

Eye Color in museum specimens of *M. janevicroy*

Country	Light Brown	Dark Brown-Black	Questionable
USA	12	2	
Mexico	34		1
El Salvador	1		
Nicaragua	5	2	
Costa Rica	5		
Venezuela		2	
Total	57	6	
